# Green Synthesis of Ag-MnO_2_ Nanoparticles using *Chelidonium majus* and *Vinca minor* Extracts and Their In Vitro Cytotoxicity

**DOI:** 10.3390/molecules25040819

**Published:** 2020-02-13

**Authors:** Alexandra Ciorîță, Maria Suciu, Sergiu Macavei, Irina Kacso, Ildiko Lung, Maria-Loredana Soran, Marcel Pârvu

**Affiliations:** 1 Babeș-Bolyai University, Faculty of Biology and Geology, 44 Republicii, 400015 Cluj-Napoca, Romania; alexandra.ciorita@itim-cj.ro (A.C.); maria.suciu@itim-cj.ro (M.S.); marcel.parvu@ubbcluj.ro (M.P.); 2 National Institute for Research and Development of Isotopic and Molecular Technologies, 400293 Cluj-Napoca, Romania; sergiu.macavei@itim-cj.ro (S.M.); irina.kacso@itim-cj.ro (I.K.); ildiko.lung@itim-cj.ro (I.L.); 3 Technical University, Faculty of Mechanical Engineering, Department of Mechatronics and Machine Dynamics, 400641 Cluj-Napoca, Romania

**Keywords:** silver-manganese oxide, nanoparticles, green synthesis, *Vinca minor*, *Chelidonium majus*

## Abstract

Medicinal plants are often used as reducing agents to prepare metal nanoparticles through green-synthesis due to natural compounds and their potential as chemotherapeutic drugs. Thus, three types of eco-friendly Ag-MnO_2_ nanoparticles (Ag-MnO_2_NPs) were synthesized using *C. majus* (CmNPs), *V. minor* (VmNPs), and a 1:1 mixture of the two extracts (MNPs). These NPs were characterized using S/TEM, EDX, XRD, and FTIR methods, and their biological activity was assessed in vitro on normal keratinocytes (HaCaT) and skin melanoma cells (A375). All synthesized NPs had manganese oxide in the middle, and silver oxide and plant extract on the exterior. The NPs had different forms (polygonal, oval, and spherical), uniformly distributed, with crystalline structures and different sizes (9.3 nm for MNPs; 10 nm for VmNPs, and 32.4 nm for CmNPs). The best results were obtained with VmNPs, which reduced the viability of A375 cells up 38.8% and had a moderate cytotoxic effect on HaCaT (46.4%) at concentrations above 500 µg/mL. At the same concentrations, CmNPs had a rather proliferative effect, whereas MNPs negatively affected both cell lines. For the first time, this paper proved the synergistic action of the combined *C. majus* and *V. minor* extracts to form small and uniformly distributed Ag-MnO_2_NPs with high potential for selective treatments.

## 1. Introduction

The need to understand how organisms function at ultrastructural levels helped filling the gap between different domains such as metallurgy, renewable energy, cosmetics, food industry, or medicine, where more often scientists look for eco-friendly alternatives in the world of bacteria, fungi, or even viruses. This facilitated the evolution of nanotechnology towards interdisciplinary applications, especially for medical purposes. In this field, the progress of nanotechnology led to the development of nanoparticles (NPs), as contrast agents or in targeted treatments. The NPs are defined as nanoscaled structures that do not exceed 100 nm in size and have physical, chemical, and biological properties that differ from those of the bulk material, primarily due to an increased surface to volume ratio of the former [1,2]. Due to their importance in clinical applications, the NPs are intensively investigated, and major progress was made in recent years on green synthesis techniques of nanomaterials and on assessing their biological effects on living organisms [3,4].

Environmentally friendly NPs’ production techniques are a challenge that made nanotechnology one of the most studied and well financed domains of the past decades. Indeed, in the last ten years, the National Nanotechnology Initiative spent more than $27 billion in the U.S. only, and the European Commission dedicates €1.1 billion to this field by the end of 2020 through HORIZON 2020 projects [5,6]. In Asia, the nanotechnology research competition between China and Japan resulted in costs that increased almost constantly by 20% each year since 2003 [7,8]. As a result of those worldwide efforts in the field of NPs, a brief search on Web of Science revealed a number of more than 2800 published articles on green synthesized nanoparticles for 2019 only [9,10].

One of the reasons why NPs are of such high interest is that they pose great potential for targeted treatment of various types of cancer. The incidence of human cancer rapidly increased throughout the world in the last years and as a consequence, the research activity for therapy has intensified [11,12]. Skin cancer (basal cell carcinoma, squamous cell carcinoma, and melanoma) is one of the most rapidly increasing types of cancer worldwide [13]. Multiple factors (i.e., radiation, medication, occupational exposure, genetics, etc.), are responsible for this high incidence, and according to World Cancer Research Fund, in 2018 more than 300,000 new cases of skin cancer were reported, with the highest rates declared in Australia, New Zealand, and Norway [14]. Melanoma is the most aggressive type with metastatic potential. In 2019, a number of 7000 people were estimated to die of melanoma, only in the United States. However, if detected on time, there are high chances of full recovery and survival [15].

Although chemotherapeutic drugs remain the treatment of choice in many malignant tumors, a major limitation for this type of drug, however, is that their action can affect perfectly healthy cells and tissues also [16]. Therefore, additional ways of treatment and discovering of new drugs are investigated [17]. Among these alternatives, NPs are the most studied cancer inhibitors, especially those based on manganese (MnNPs) and silver (AgNPs). MnNPs pose a great interest for biological applications and could be obtained by chemical synthesis [18]. However, the MnNPs obtained in this way (e.g., Mn_2_O_3_ NPs) show negative effects in animals, such as a decrease on the production of sex hormones, as shown in rats by Negahdary et al. [19]. A more eco-friendly method for MnNPs preparation is green synthesis using plant extracts, such as those obtained from *Ananas comosus* [20], *Kalopanax pictus* [21], or *Simarouba glauca* [22]. The effects of silver NPs have been thoroughly documented in the last decades [10,23], not only for the environmental impact that it has, but also for the anticancer potential. Thus, previous studies demonstrated the inhibitory effect of green synthesized AgNPs on breast cancer (MCF-7) [24], lung epithelial adenocarcinoma (A549) [25], colon cancer (HCT116) [26], hepatocellular carcinoma (HepG-2) [27], and gastric cancer (AGS) [28] cell lines. In addition, these studies revealed that AgNPs do not have toxic effects on the cell lines at certain concentrations (between 1 and 200 µg/mL).

Besides the NPs made of manganese and silver exclusively, Ag-MnO_2_ combination attracted a significant interest [29,30]. As an example concerning the positive effects of combining silver and manganese in such a complex, a publication reported that free silver ions have negative effects on several human cell lines, and by combination with MnO_2_, the silver ions were stabilized, thus reducing their toxicity [31].

However, the process of choosing the right plant for extract preparation and NPs synthesis is usually based on the economic importance that the plant has. Thus, medicinal herbs are considered a promising source for new drugs development that aims for the prevention and treatment of cancer. Natural compounds are studied intensively in this regard, and major progress was made in order to determine the chemical structure of the molecules and the mechanisms of action [32,33]. Among these compounds, alkaloids, flavonoids, lignans, saponins, terpenes, or taxanes play significant roles in cancer therapies [17]. Hence, we focused our experiments on *Chelidonium majus* L. (celandine) and *Vinca minor* L. (lesser periwinkle) due to their high content of natural products, especially alkaloids, which are important agents in pharmacologically active drugs used in cancer therapies. Both species have rather complex chemical compositions with two different types of alkaloids, i.e., isoquinoline alkaloids for *C. majus*, such as sanguinarine, chelidonine, or chelerythrine [34,35,36], and terpene indole alkaloids for *V. minor*, such as vincamine [37,38]. The antitumor activity of these two plants has been thoroughly studied before in many in vitro and in vivo experiments [39,40,41,42], moreover, three *Vinca* alkaloids (vincristine, vinblastine, and vinorelbine) have been approved for use in cancer therapies in the United States [43].

The aim of this work was to obtain a green synthesized Ag-Mn nanocomplex with enhanced properties provided by the nanomaterials and the two plant extracts used individually and in combination, and to test that nanocomplex concerning its potential as topical treatments of melanoma without affecting the normal cells, such as keratinocytes. Based on the available scientific literature, as far as we are aware, this approach has never been considered before in other studies. *C. majus* was previously used for NPs preparation [44], but to our knowledge, there are no studies in this direction for *V. minor*. In this regard, *C. majus* and *V. minor* hydroalcoholic extracts were used to prepare three types of Ag-MnO_2_ NPs, i.e., CmNPs (prepared using only *C. majus* extract), VmNPs (prepared using only *V. minor* extract), and MNPs (prepared using a mix of the two extracts in a 1:1 ratio). Those three NP types were then tested for their antitumor/cytotoxic effects on human skin melanoma (A375) and normal human keratinocytes (HaCaT) cell lines.

## 2. Results and Discussions

### 2.1. Plant Extracts

**Figure 1:**
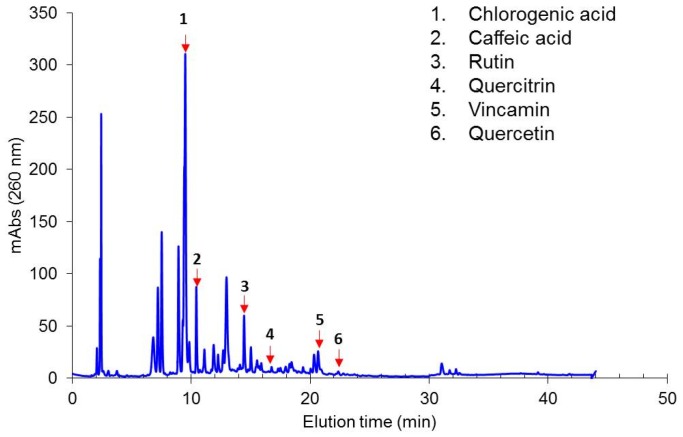
HPLC-DAD chromatogram showing the chemical composition of the *V. minor* plant extract prepared in this study.

*C. majus* plant extract was previously characterized and had chelidonine and berberine alkaloids, polyphenols, and sterols in its composition [45]. Using the HPLC-DAD method the chemical composition of *V. minor* extract was also determined in this work. The results showed that the prepared plant extract contained chlorogenic acid as the main component and other secondary metabolites, the vincamine alkaloid being one of these. (Figure 1).

### 2.2. Nanoparticles

#### 2.2.1. S/TEM Analysis

**Figure 2:**
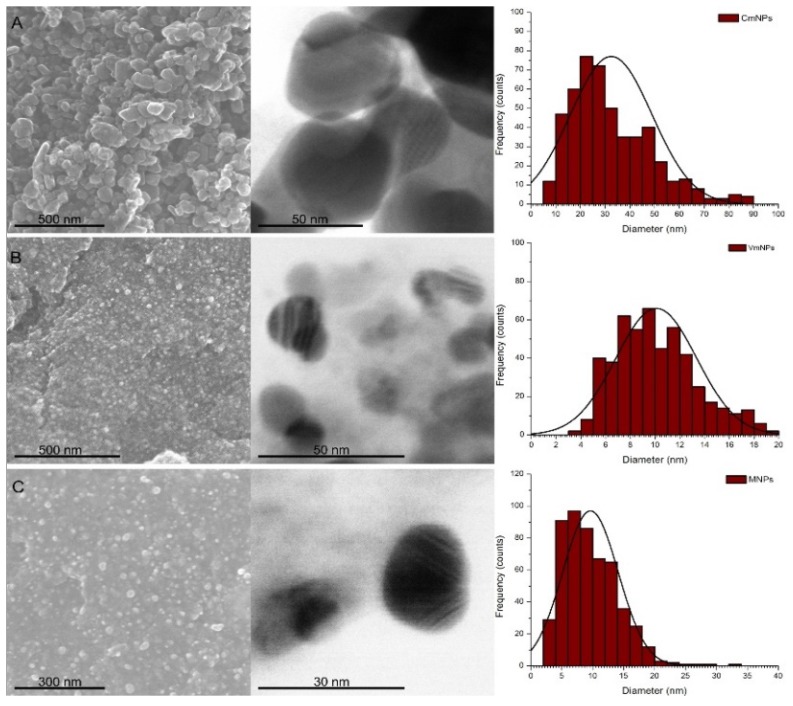
SEM and TEM micrographs showing the morphology of nanoparticles and the graphs with Gaussian fit of the size distribution; **A**: CmNPs, **B**: VmNPs, **C**: MNPs.

The NPs obtained in this study were with various forms (polygonal, oval, and spherical). The size distribution of those NPs varied in relation with the plant extract that was used. Thus, CmNPs had an average size of 32.47 nm ± 0.73 nm (mean ± S.E.M.), VmNPs had an average size of 10.09 nm ± 0.14 nm, and MNPs had an average size of 9.36 nm ± 0.19 nm (Figure 2).

**Figure 3:**
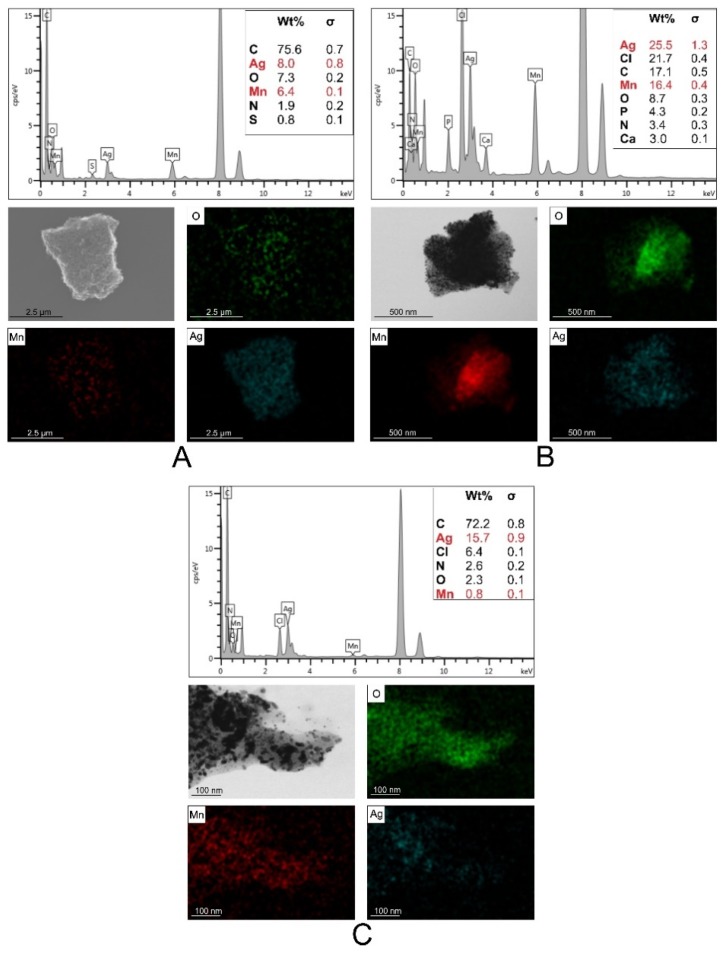
EDX spectra showing the elemental distribution in the green synthesized nanoparticles; **A**: CmNPs, **B**: VmNPs, **C**: MNPs.

According to the elemental distribution as shown by the EDX analysis, the NPs formed were core-shells, with manganese oxide cover by silver oxide, and an additional layer of organic compound formed by the plant extracts. CmNPs and VmNPs had a balanced weight ratio between manganese and silver elements, whereas MNPs had a lower content of manganese as compared to the former two (Figure 3).

#### 2.2.2. XRD Analysis

**Figure 4:**
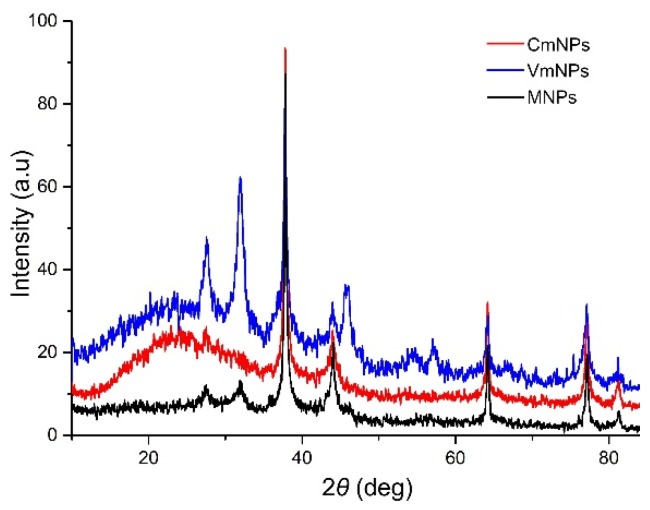
XRD pattern of resulted Ag-MnO_2_ NPs.

The crystalline nature of the resulted nanoparticles was confirmed by XRD analysis (Figure 4). All three samples showed four distinct diffraction peaks at 2θ values that corresponded to the reflection planes of (111), (200), (220), and (311) characteristic to the face centered cubic structure of silver, and (110), (101), (211) for MnO_2_ (reference files PDF card no. 03-065-8428 and PDF card no. 00-001-0799). For silver, the lattice constants were a = 4.09788(16) Å, b = 4.09788(16) Å, and c = 4.09788(16) Å. For manganese, the lattice constants were a = 3.945(15) Å, b = 3.945(15) Å, and c = 2.86(2) Å.

The crystallite size was also determined using the Williamson-Hall method. Thus, for silver the crystallite size was 69(23) Å, and for manganese was 11.953230 Å. Our results are consistent with previous findings [1,25,30,31,46,47,48]. However, additional peaks were observed, and they could be associated to Ag_2_O at 2θ values of 27°, 32°, and 44° (PDF card no. 00-041-1104) and to the organic compounds from the plant extracts, which were responsible for metal ions reduction and stabilization of the NPs. Diffraction patterns indicated that all three samples had a crystalline nature, and that the Ag-MnO_2_ complex was successfully formed. From those perspectives, the NPs synthesized in the present study are similar with the Ag-MnO_2_ nanostructures obtained by others in previous studies [49,50].

#### 2.2.3. FTIR Analysis

The absorption bands identified in the spectra of the nanoparticles, functionalized with the *C. majus*, *V. minor*, and the mixed extracts, can be assigned to the characteristic functional groups of the organic compounds from the natural extracts used and to the metal-oxygen vibrations in nano-powder. In our opinion, the width of the vibration bands is due to the complexity of the natural extracts that we used, where compounds such as flavonoids, polyphenols, organic acids, carotenoids, alkaloids were present. In the FTIR spectrum of CmNPs, VmNPs, and MNPs, the characteristic stretching vibration bands of O-H around 3400 cm^−1^ and of C-H at 2915 and 2846 cm^−1^ appeared, but were irrelevant because they are specific for the majority of bioactive compounds [51,52].

**Figure 5:**
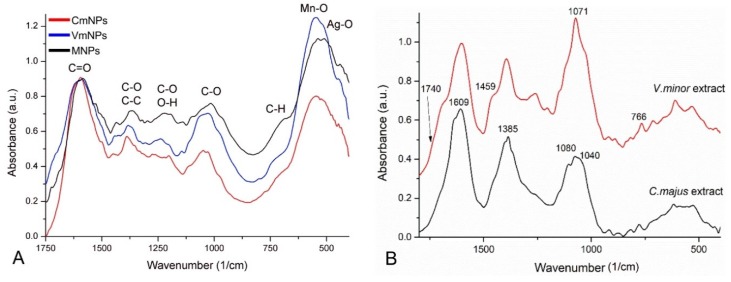
FTIR spectra showing the characteristic stretching vibration bands of the obtained Ag-MnO_2_ NPs (**A**), and of the plant extracts (**B**) used for this study.

In our case, the important FTIR domain was 1750–500 cm^−1^. In the 1750–1580 cm^−1^ spectral range, the stretching vibration of C=O group, from aldehydes and ketones, stretching vibrations of C=C and bending vibration of N-H groups, from amino-acids, appeared [44,52,53]. For CmNPs and VmNPs, the stretching vibration of C-O and bending of C-H groups were identified between 1450–1300 cm^−1^, at 1380 cm^−1^, and slightly shifted at 1365 cm^−1^ for MNPs [44]. In the spectral domain 1270–1150 cm^−1^, the stretching vibrations of C-O and N-H, and bending vibrations of O-H groups were identified [54]. The characteristic stretching vibration of Mn-O appears as a broad and intense vibration band in the 600–450 cm^−1^ spectral range (Figure 5A) [55,56,57]. The Ag-Ag metallic bond vibrations appear below 400 cm^−1^ [58], thus, they cannot be observed in the measured spectral domain. The main alkaloid of *V. minor* is vincamine, and the FTIR spectra of this alkaloid has strong characteristic vibrational bands at 1742, 1458, 1074, and 745 cm^−1^ [58]. These bands were also identified in the *V. minor* extract that was prepared in this study, hence at 1742 cm^−1^ and 1459 cm^−1^, as a shoulder, and 1071 cm^−1^ and 766 cm^−1^, respectively (Figure 5B). The same bands were also present as shoulders in the spectra of the VmNPs.

Berberine from *C. majus* extract presents a strong band of aromatic vibrations at 1609 cm-^1^, the CH wag vibrations around 1385 cm^−1^, the ring deformation band at 1080 cm^−1^, and the OCO stretching of dioxolan ring at 1040 cm^−1^ as a shoulder (Figure 5B) [59]. On the spectra of CmNPs all these bands were also present, slightly shifted or as shoulders (Figure 5A).

### 2.3. Cell Toxicity

#### 2.3.1. MTT Assay

**Figure 6:**
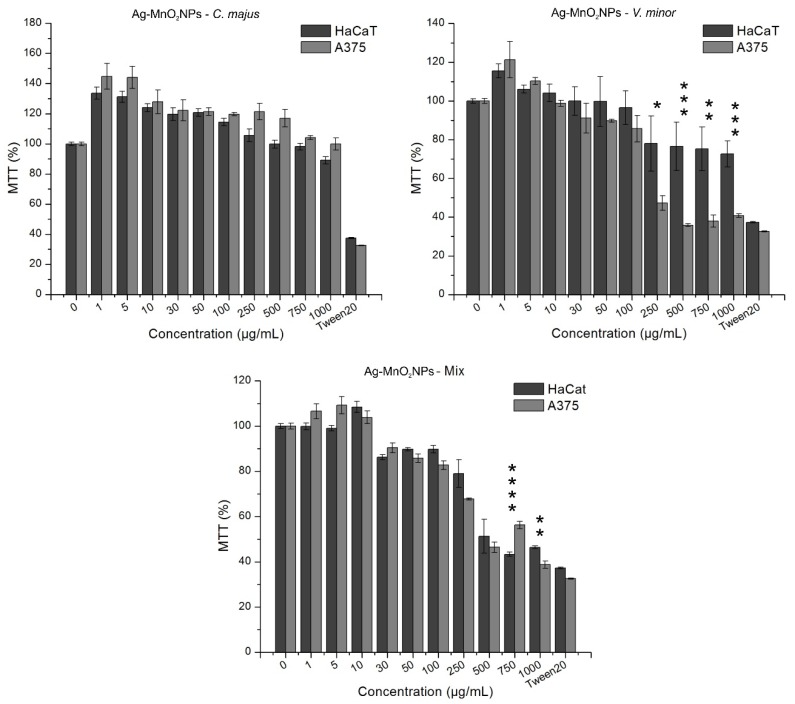
In vitro cytotoxic effect of green synthesized Ag-MnO_2_ NPs on normal keratinocytes (HaCaT) and skin melanoma (A375) compared to a positive control (untreated cells) and a negative control (cells treated with 2% Tween20); ****: *p* < 0.0001, ***: *p* < 0.005, **: *p* < 0.05, *: *p* < 0.1.

In Figure 6, MTT activities of HaCaT and A375 cells treated with green synthesized nanoparticles are depicted.

According to ISO 10993-5, cell viability results can be interpreted as following: >80% no cytotoxicity, 80–60% weak cytotoxicity, 60–40% moderate cytotoxicity, and <40% strong cytotoxicity [60]. When NP treatment was applied, the cell viability decreased in a dose-dependent manner for both cell lines and in relation with the NP type used as treatment. Thus, when treated with CmNPs, HaCaT cells expressed a viability which was above that of the untreated control (100%) for samples incubated with 1 µg/mL (133.5%) to 250 µg/mL (105.65%), and decreased in a dose dependent manner down to 89.07% at 1000 µg/mL. Whereas, a similar trend was observed with respect to A375 cells, nine out of the ten tested concentrations induced mitochondrial activity responses above untreated control (144.8% for 1 µg/mL, down to 104.11% for 750 µg/mL). These results could indicate that CmNPs had a proliferative effect on the cell types that we used, which was also observed by Moulton et al. when green synthesized AgNPs were used in tests on HaCaT cells. The highest NP concentration used by the mentioned authors was 100 µg/mL, and that concentration did not induce any cytotoxicity on those cells [61]. Although we exceeded that concentration by a 10-fold, no cytotoxic effect was observed on normal keratinocytes. A similar result was obtained for A375, which is consistent with the results of other studies where AgNPs were used [62]. As the findings of others suggested that large nanoparticles have no cytotoxic effect on cells [63], this lack of toxicity in our study could be explained by the NPs’ size rather than the surface coating or the plant extract used.

**Figure 7:**
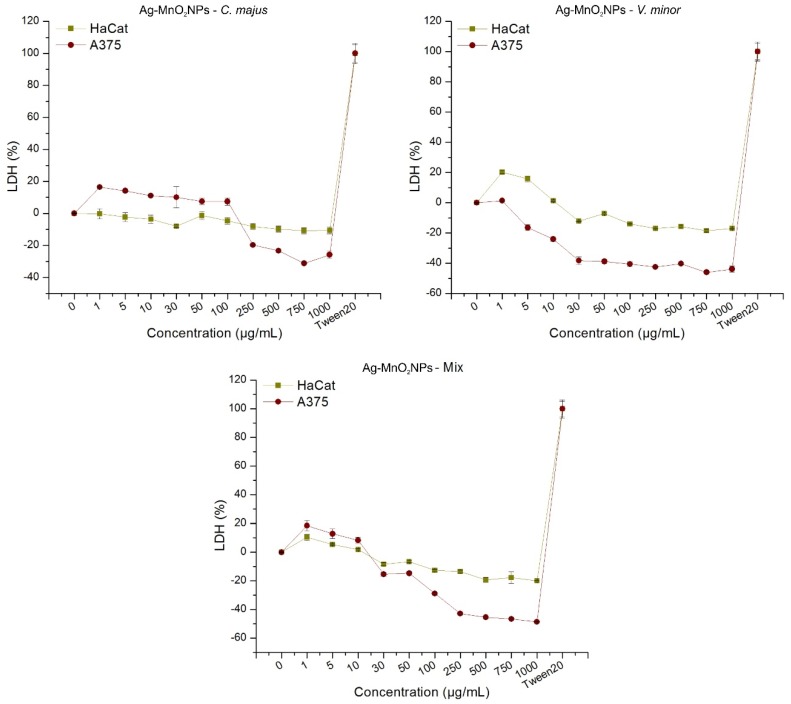
In vitro analysis of LDH release on normal keratinocytes (HaCaT) and skin melanoma (A375) treated with green synthesized Ag-MnO_2_NPs using the three plant extract formulations.

When treated with VmNPs, HaCaT cells showed weak cytotoxicity (78.04%, 76.56%, 75.28%, and 72.67%) at high concentrations (250, 500, 750, and 1000 µg/mL, respectively) and at the same concentrations, A375 cells showed moderate (47.25% at 250 µg/mL and 40.77% at 1000 µg/mL) and strong (35.87% at 500 µg/mL and 37.92% at 750 µg/mL) cytotoxicity (Figure 7). Low to no cytotoxicity on HaCaT was observed by other researchers also [64,65], and negative effect on A375 cells produced by green synthesized AgNPs was reported before [66,67]. In our study, A375 cells were significantly (*p* < 0.0001) more affected than HaCaT cells by VmNPs concentrations, with the lowest viability value (35.87%) registered at 500 µg/mL. Consistent with our results, other studies also reported that HaCaT cells were less sensitive than cancer cells when exposed to AgNPs [68,69], and the cytotoxic effect induced in cancer cells in our study could be due to the chemical composition of the plant extract that we used [70]. Vincamine is the main alkaloid of *V. minor* that has medicinal value because of the indole structure known for antimitotic activity, making it a possible drug for cancer therapy [71,72]. In addition, the mechanical resistance to deformation (or bending stiffness, [73]) of the cells plays an important role in NP internalization. Thus, cancerous cells typically have a lower stiffness (they are also defined as ‘soft’) when compared to the tumor tissue that they form and to normal cell lines, property which allows them to migrate and eventually metastasize [74,75]. The reported bending stiffness is 100 kPa for HaCaT cells [76], and varies from 0.76 kPa [77] to 3.9 kPa [78] for A375 cells depending on the experimental setup [79,80]. Considering those stiffness values, the stronger cytotoxicity that we observed on A375 compared to HaCaT could be explained by the high capacity of A375 to uptake larger amounts of nanoparticles due to their lower stiffness and regardless of the physical properties of the NPs. Moreover, as soon as the NPs were internalized, the organic compound formed by vincamine, rutin, quercetin, and other natural products could have helped generating cytotoxicity. However, further analyses are required to statistically determine if A375 cells are able to internalize more VmNPs than HaCaT cells.

MNPs negatively affected both cell lines at high concentrations. HaCaT cells registered the lowest viability value at 750 µg/mL (43.35% viability) as compared to A375 cells, which had 56.31% viability at the same concentration, the statistical analysis indicating that HaCaT cells were significantly (*p* < 0.0001) more affected than A375 cells at this concentration (Figure 7). The lowest viability value was registered at 1000 µg/mL for A375 (38.8% viability), and compared to HaCaT at the same concentration (46.45% viability), the difference was significant (*p* < 0.05), indicating that A375 cells were more affected by MNPs at 1000 µg/mL. Similar negative effect on HaCaT viability was reported for PEG-coated (polyethylene glycol) [81,82] and naked AgNPs [83,84] in dose- and time-dependent manners. The cytotoxic effect on both cell lines could be explained by three factors. First, MNPs were significantly smaller than the other two types of NPs synthesized, and the chance of cytotoxic response increases once the NP size is under 10 nm [85]. The exact response of the cells to NP size is yet insufficiently studied; however, three general opinions are accepted: 1) The surface charge of the NPs strongly influences the minimal size preference, 2) NP endocytosis is size-dependent, and 3) passive uptake as method of internalization is preferred [86]. Second, based on XRD patterns observed and EDX analyses used in the present study, the amount of MnO_2_ was lower in MNPs than on CmNPs and VmNPs, and according to Krishnaraj et al., MnO_2_ reduces the toxicity of free Ag ions when both are combined [48]. Third, the combination of the two extracts could have led to the accumulation of a higher content of alkaloids and other phenolic compounds. As such compounds are known for cytotoxic activity [36,37,38], their internalization by keratinocytes along with the NPs could thus explain the high cytotoxicity of MNPs observed also on HaCaT and not just on A375 cells.

#### 2.3.2. LDH Assay

In Figure 7, the LDH release from HaCaT and A375 cells treated with the three green synthesized NPs is depicted.

The LDH release in cell media is an indicator of cells’ status and, depending on the formulae used for calculation, one can determine the extent of cell growth inhibition or necrosis [60]. The two cell lines that we tested reacted differently to the treatment with the three types of NPs synthesized in this study. When treated with CmNPs, HaCaT cells had a relatively constant LDH release at all tested concentrations, which, as confirmed by χ^2^ test (*p* = 1, SD = 0.03), was comparable with that observed in the untreated controls. In contrast with HaCaT cells, A375 cells showed a dose dependent response. Thus, at low concentrations (1–100 µg/mL), LDH release increased by up to 16.43% as compared to untreated control, and decreased consistently by ~30% for all the largest concentrations (250–1000 µg/mL). The extent of damages induced on A375 were significantly higher (*p* < 0.0001) as compared to those induced in HaCaT at high concentrations.

VmNPs affected the HaCaT cells in a dose dependent manner, where LDH release was by up to 20.28% higher than in untreated controls, for 1 µg/mL, gradually decreased by up to 12.19% smaller than in the controls for the 30 µg/mL, and then remained constant at that value for all the remaining concentrations (30–1000 µg/mL, *p* > 0.05). A similar pattern was observed on A375 cells, and when the results were compared to HaCaT cells, melanoma cells were significantly more affected: Around −40% LDH release for the 30–1000 µg/mL (*p* < 0.0001; Figure 7).

When treated with MNPs, both cell lines also responded in a dose-dependent manner. Keratinocyte LDH release was slightly increased (10.39%) at the lowest concentration, decreasing slowly and gradually to −10%, from where it remained constant for the remaining concentrations (Figure 7). The effect seemed enhanced for the A375 cells: Higher LDH release at small concentrations (18.51% at 1 µg/mL) and much lower LDH release at larger concentrations (−48.65% at 1000 µg/mL).

Habas et al. revealed an increase in LDH concentration in a dose-dependent manner [87], and Garvey et al. observed no relation between LDH release and NP concentration, concluding that the assay could have interacted with the NPs [68].

When an LDH assay is performed with respect to the NP cytotoxicity, several factors should be considered in order to eliminate the false positive or false negative results. AgNPs could inactivate LDH in a dose dependent manner [88], but in our experiment the vehicle controls showed no differences compared to positive and negative controls. LDH is limited by the number of available cells. Thus, when the majority of cells are dead or the membranes are blocked by the NPs, no LDH could be released, and increased NP concentrations will decrease the LDH through adsorption by endocytosis [88]. Since our controls showed no difference between batches or concentrations, we could conclude that at low concentrations, cell membranes were blocked by the NPs, and at high concentrations, the majority of cells were dead or in the process of dying by apoptosis.

#### 2.3.3. NO Assay

**Figure 8:**
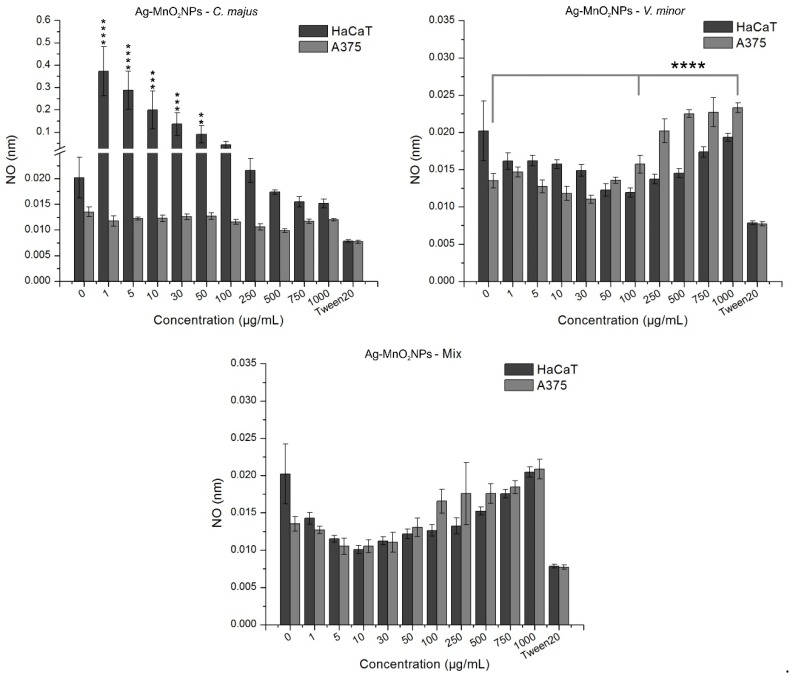
In vitro analysis of the NO release by normal keratinocytes (HaCaT) and skin melanoma (A375) cells treated with green synthesized Ag-MnO_2_NPs using the three plant extract formulations; ****: *p* < 0.0001, ***: *p* < 0.005, **: *p* < 0.05.

In Figure 8, the NO release by HaCaT and A375 cells treated with green synthesized nanoparticles is depicted. At all tested concentrations, A375 cells treated with CmNPs had a constant release of NO, which did not differ to that of the untreated controls, as indicated by the χ^2^ test (*p* = 1, SD = 0.0008). HaCaT cells had a dose-dependent response to CmNPs, with a significantly higher NO release (up to 0.35 nM compared to 0.02 nM for the untreated controls) at low concentrations (1–250 µg/mL, *p* < 0.05). Cells treated with VmNPs were differently affected by the nanoparticles. The highest NO release for HaCaT was reached at 1000 µg/mL, but when compared to untreated control, the value was within normal variations as shown by statistical analyses. A375 had significantly higher NO release (*p* = 0.0001) at high concentrations compared to control, indicating oxidative stress for melanoma cells compared to normal keratinocytes. For MNPs, HaCaT had a dose-dependent response to the nanoparticles. ANOVA test indicated a difference between concentrations, but no significant difference was observed when the results were compared to untreated control (*p* = 1). NO release for A375 was low from 1–50 µg/mL, with no significant statistical difference between treated samples and control. Starting with 100 µg/mL, the NO release increased, and compared to control, results showed a moderate statistical significance (*p* < 0.05), proving that A375 was affected at concentrations above 100 µg/mL, whereas HaCaT was not negatively affected (Figure 8).

Cytotoxicity could be induced by increased oxidative stress and generation of reactive oxygen species (ROS) [89]. However, NO was shown to play a rather inconstant role in biological regulation because of the contradictory effects since it could have both pro-apoptotic and anti-apoptotic actions. Thus, low doses could inhibit apoptosis development, but also inhibit tumor proliferation inducing apoptosis in tumor cells [90].

HaCaT treated with CmNPs showed high mitochondrial activity through MTT assay at low concentrations, and results could be correlated with NO assay that showed high values at same NP concentrations. Since LDH release was almost constant, we could conclude that the results were associated with a high number of cells and in this case, CmNPs had a proliferative effect on the keratinocytes. A375 treated with CmNPs registered a decrease in cell viability in a dose-dependent manner. The values were not associated with cell toxicity according to MTT assay, but a correlation was observed between LDH and NO assays, where at low concentrations LDH had high concentrations and the NO results in this case could be associated with tumor cell inhibition, meaning that A375 were negatively affected compared to HaCaT.

HaCaT treated with VmNPs registered a decrease in viability from low to higher NP concentrations according to the MTT assay. The values at low concentrations were above the positive control, and this explains the higher LDH values registered, since NO concentrations were low, concluding that HaCaT were not affected by these nanoparticles. On the other hand, significant differences were observed on A375 treated with VmNPs at high concentrations.

All three assays showed a decrease in cell viability associated with apoptotic, but not necrotic events, indicating that A375 were more affected than HaCaT. Both cell lines treated with MNPs were negatively affected at high concentrations. Cell viability reached values under 50% for both HaCaT and A375, with slightly higher values for HaCaT. LDH concentrations could be associated with cell death by apoptosis and NO release with oxidative and anti-proliferative response to the NPs used.

### 2.4. Nanoparticle Uptake by HaCaT and A375 Cells

**Figure 9:**
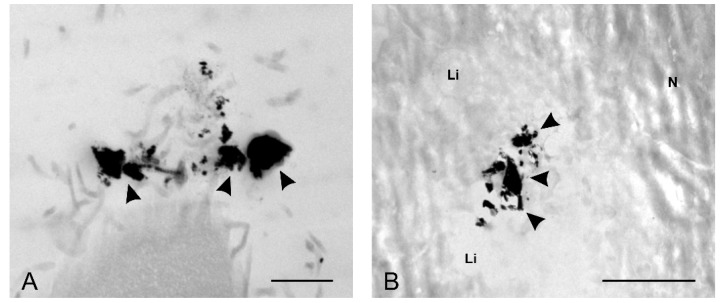
TEM micrographs showing the presence of electron-dense structures (black arrow heads) along the membranes of A375 cells (**A**, with bar at 1 µm) and on the surface of HaCaT cells (**B**, bar at 2 µm); Li = lysosome, N = nucleus.

**Figure 10:**
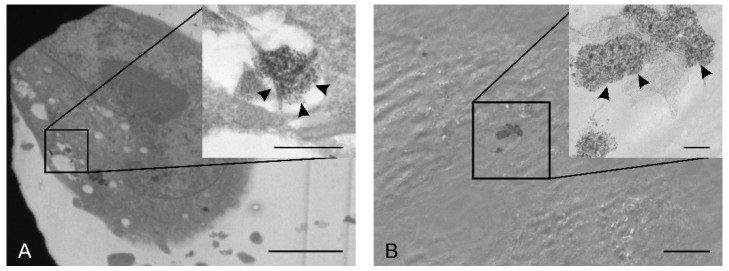
TEM micrographs showing the presence of electron-dense structures (black arrow heads) inside A375 cells (**A**, bar at 5 µm, with inset with bar at 500 nm) and HaCaT cells (**B**, bar at 2 µm, with inset with bar at 200 nm).

TEM analyses were conducted in order to determine if the obtained NPs could penetrate the cells in 24 h. Due to their larger size and because larger NPs are less likely to be uptaken by the cells [86], only CmNPs were tested. Thus, we could observe CmNPs along the membranes of A375 and of HaCaT cells (Figure 9), and the smaller ones were also found inside cells (Figure 10).

**Figure 11:**
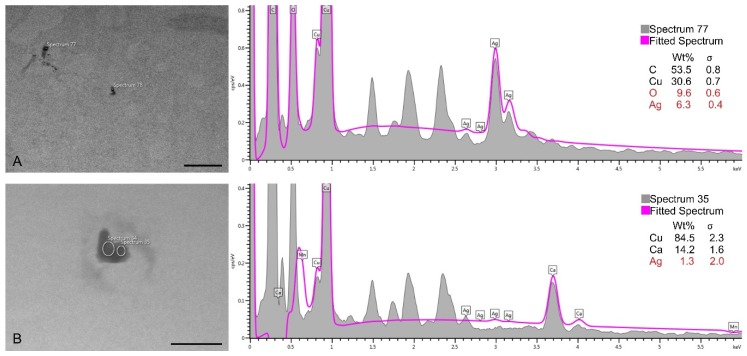
TEM micrographs of the tested cells and EDX spectra confirming the presence of silver in the electron-dense accumulations observed inside A375 cells (**A**, bar at 1 µm) and HaCaT cells (**B**, bar at 250 nm).

The EDX analysis confirmed the presence of silver in the electron-dense accumulations identified inside the cells. Since silver had a slightly higher concentration than MnO_2_ in CmNPs (please see Figure 3A), it was easier to detect (Figure 11).

As it happens, in the case of cytotoxicity, cellular uptake also depends on the size of NPs, besides their shape, material, or surface charge [91,92]. TEM analysis showed that smaller CmNPs were able to penetrate the cells. However, the specific means through which the NPs were able to enter are yet to be determined. Further analyses are required at different time rates, in order to catch the exact moment of penetration and to determine the type of uptake (active, by endocytosis, or passive, by diffusion). Based on experimental setups (exposure time, concentration), in vitro analyses with different or same types of cell, showed how they react differently to the same type of NPs [93]. That being so, using silica gold core-shell NPs conjugated with folic acid, Majidi et al. [94] were able to quantitatively determine the intracellular uptake of gold, by A375 cell lines, through inductively coupled plasma method. Additionally, Andersson et al. [85] showed how lung epithelial cells preferentially uptake smaller TiO_2_ nanostructures, and Arora et al. [83] demonstrated that HaCaT cells were able to uptake larger amounts of AgNPs after the cells were exposed to UVB-irradiation.

Therefore, various factors contribute to the cytotoxicity induced by NPs in vitro, and to exactly determine why and how it happens, further investigations are required. Other important aspects that need to be taken into account are the incomparable responses generated in vitro and in vivo, where multiple systems and organs interact and could lead to inconsistencies between results. However, to our knowledge, the capacity of A375 and HaCaT cells to uptake green synthesized Ag-MnO_2_ NPs had not been previously investigated, and in a future perspective this subject could bring valuable insights.

## 3. Materials and Methods

### 3.1. Plant Material

*C. majus* and *V. minor* were collected from ‘Alexandru Borza’ Botanical Garden of Cluj-Napoca (46°45′36′’N and 23°35′13′’E), Romania. Voucher specimens (CL 663 692 for *C. majus*, and CL 665 977 for *V. minor*) were deposited at the Herbarium of ‘Babeș-Bolyai’ University of Cluj-Napoca.

### 3.2. Hydroalcoholic Extract Preparation

For both species, fresh herba (285 g of *C. majus* and 260 g of *V. minor*) was extracted with 60% 500 mL ethanol (Merck, Bucharest, Romania) by the cold repercolation method at room temperature, for three days. *C. majus* extract contained 1 g plant material in 1 mL 30% ethanol (*w*/*v* ratio), and *V. minor* extract contained 1 g plant material in 1.2 mL 30% ethanol (*w*/*v*). The plant extracts were stored at 4 °C until further use.

### 3.3. Chemical Composition of Plant Extracts

The chemical composition of *C. majus* extract was determined as previously described, i.e., by high-performance liquid chromatography method coupled with mass spectrometry (HPLC-MS; Agilent Technology, Santa Clara, CA, USA) [45]. In this study, *V. minor* extract was characterized using a HPLC-DAD approach (Agilent Technology, Waldbronn, Germany), as described by Andreicut et al. [95]. A Nucleosil 100 C18 column (240 mm × 4.6 mm, and 5 µm particle size) was used for chromatographic separation, from Macherey-Nagel (Duren, Germany). The injection volume was 5 µL of *V. minor* extract at a column temperature of 25 °C and a flow rate of 1.2 mL/min. Ammonium acetate (purity ≥98%, Sigma-Aldrich, Merck KGaA, Darmstadt, Germany) of 10 mM concentration and pH 5 was used as solvent A, and acetonitrile (Sigma-Aldrich, Merck KGaA, Darmstadt, Germany) as solvent B. The gradient variations were the following: 0 to 15 min, from 8% to 30%; 15 to 25 min, isocratic at 30%; 25 to 35 min, from 30% to 85%; 35 to 38 min, from 85% to 95%; 38 to 39 min, isocratic at 95%; and 39 to 39.1 min, at 8% and kept there until 40 min. The standards used for calibration were chlorogenic acid, caffeic acid, vincamine, vinblastine, rutin, quercitrin, and quercetin, acquired from Sigma-Aldrich (Merck KGaA, Darmstadt, Germany). The DAD detector measured the spectrum from 210 to 700 nm, while the chromatogram was monitored 220, 280, 340, and 425 nm.

### 3.4. Synthesis of Ag-MnO_2_ Nanoparticles

Ag-MnO_2_ NPs preparation was performed in two stages. First, MnO_2_ NPs were obtained by mixing 200 mg of KMnO_4_ (VWR Life Science Amresco, LLC, Ohio, USA), with 16 mL ultrapure water (Direct-Q® 3 UV Water Purification System, Merck, Germany ) and 8 mL plant extract. The solution was sonicated for 1 h in a sonication bath (TranssonicT 470/H, Elma, Singen, Germany), and the formation of NPs was indicated by the change in color from purple to dark brown. The NPs were washed with water and ethanol by repeated centrifugations at 7000 rpm and dried at 60 °C for 24 h. Second, the Ag-MnO_2_ NPs were obtained by magnetic stirring of MnO_2_ NPs with 5 mM AgNO_3_ (VWR International GmbH, Wien, Austria) solution (MnO_2_ NPs: AgNO_3_ solution = 0.75 g/mL) in plant extract (AgNO_3_ solution: plant extract = 1:2, *v*/*v*), for 6 h at 1000 rpm and room temperature. The NPs were washed and dried like in the first step.

Three types of NPs were obtained using the plant extracts individually and a mixture of extracts in 1:1 (v/v) ratio. Those three types were:

(1) CmNPs—prepared using only *C. majus* extract;
(2) VmNPs—prepared using only *V. minor* extract;
(3) MNPs—prepared using a mix of the two extracts in 1:1 ratio.

### 3.5. Characterization of Ag-MnO_2_ Nanoparticles

The Ag-MnO_2_ nanoparticles obtained were characterized through scanning-transmission electron microscopy (S/TEM), X-ray diffraction (XRD), and Fourier-transformed infrared spectroscopy (FTIR). S/TEM was performed using S/TEM HITACHI HD2700 cold field emission, operated at 200 kV (HITACHI, Tokyo, Japan), and coupled with EDX (Oxford Instruments, Oxford, UK, AZtec Software, version 3.3) used for elemental detection. The size distribution of nanoparticles was determined using Image J software (version Java 8). XRD analysis was recorded using high resolution SmartLab X-ray diffractometer (Rigaku, Tokyo, Japan) operated at 9 kW and coupled with SmartLab Guidance software (SmartLab Studio II package software). FTIR measurements were performed with a JASCO 6100 FTIR spectrometer (JASCO Deutschland GmbH, Pfungstadt, Germany) in the 4000–400 cm^−1^ spectral domain with a resolution of 4 cm^−1^ by using the KBr (for IR spectroscopy, Merck KGaA, Darmstadt, Germany) pellet technique.

### 3.6. Cell Toxicity

#### 3.6.1. Established Cell Lines

Cytotoxicity assays were conducted on human keratinocytes (HaCaT, CLS-300493, Eppelheim, Germany), and skin melanoma (A375, ATCC CRL-1619, Wesel, Germany). The cells were cultured on 25 cm^2^ plastic dishes in Dulbecco’s Modified Eagle’s medium (DMEM) with 4.5 g/L glucose, supplemented with 10% fetal calf serum (FCS), 1% penicillin-streptomycin, and 1% L-glutamine (all products acquired from Sigma Aldrich, Merck KGaA, Darmstadt, Germany). Cells were kept in a humidified incubator at 37 °C with 5% CO_2_ atmosphere. At 80% confluence, cells were harvested using 0.25% trypsin and sub-cultured in 96 wells plates.

A 100 µL aliquot of the prepared cells were plated in each well, A375 at a density of 10^4^ cells/well, and HaCaT at a density of 12·× 10^3^ cells/well, and were allowed to attach for 24 h. After incubation, the culture medium was replaced with medium containing NPs in final concentrations ranging from 1 to 1000 µg/mL. The plates were incubated for an additional 24 h and were further analyzed for viability and oxidative stress by 3-(4,5-Dimethylthiazol-2-yl)-2,5Diphenyltetrazolium Bromide (MTT, Sigma Aldrich, Merck KGaA, Darmstadt, Germany), lactate dehydrogenase (LDH), and nitric oxide (NO) release.

#### 3.6.2. Cell Viability Assay

After 24 h incubation with the three types of nanoparticles, mitochondrial activity was assessed by MTT method. MTT solution (100 µL) was added to each well in a final concentration of 0.5 mg/mL and further left for incubation for an additional 1.5 h. The resulted formazan was diluted in acidified iso-propanol and the absorbance was measured at 550 nm using BioTek Epoch plate reader (BioTek Instruments, Winooski, VT, USA) and Gen5 software (version 1.09).

#### 3.6.3. Membrane Integrity Assay

The LDH method was conducted as described by Macavei et al. [60]. Shortly, 50 μL of culture media was subtracted from test plates and together with 50 μL lithium lactate solution of 50 mM, 50 μL tris solution of 200 mM, and 50 μL of NAD solution (a mixture of ionitrotetrazolium violet, phenazine metosulphate, and nicotinamide dinucleotide; Sigma Aldrich, Merck KGaA, Darmstadt, Germany) were added to a new 96 wells plate. After 10 min, the absorbance at 490 nm and the background at 690 nm were read using BioTek Epoch plate reader and Gen5 software.

#### 3.6.4. Nitric Oxide Assay

Determination of nitric oxide (NO) concentration was done by Griess reaction. In short, NO-derived nitrosating agent (N_2_O_3_) generated by the autooxidation of NO reacts to sulphanilamide to produce a diazonium ion that is further coupled to N-(-1-napthyl)-ethylenediamine (N1-NAP) to form a product that absorbs strongly at 540 nm [96]. 50 μL of culture media was mixed with sulphanilamide. The solution was kept in dark at room temperature for 10 min. Further, N1-NAP was added and the reaction was kept in the same conditions (all reagents were acquired from Sigma Aldrich, Merck KGaA, Darmstadt, Germany). After an additional time-interval of 10 min, samples were read at 540 nm using BioTek Epoch plate reader and Gen5 software.

### 3.7. Nanoparticle Uptake by HaCaT and A375 Cells

In order to evaluate the ability of the obtained NPs to penetrate the HaCaT and A375 cells, transmission electron microscopy (TEM) analyses were performed. Fresh cell cultures were left to attach on glass slides for 24 h, at a confluence of 3·× 10^4^ cells/glass slide. Next, the solution of NPs was added. Because larger nanoparticles are considered to be less likely to be uptaken by the cells [86], only CmNPs were tested at 1000 µg/mL concentration. The cells were incubated with CmNPs for an additional 24 h in the same experimental setups.

After this period, the cells were fixed with 2.7% glutaraldehyde for 1 h. Samples were then washed four times in PBS (phosphate buffer saline), fixed with osmium tetroxide (OsO_4_) for another hour, and washed again four times with PBS. After fixation, the samples were placed in acetone at different concentrations for gradual dehydration, afterwards infiltrated and included in Epon 812 epoxy resin, and then polymerized at 60 °C (all reagents were acquired from Sigma Aldrich, Merck KGaA, Darmstadt, Germany). The formed blocks were modelled, and ultrathin sections of 70 nm were obtained using Leica UC7 ultramicrotome (Leica Microsystems, Wetzlar, Germany) and a Diatome diamond knife (DiATOME, Hatfield, PA, USA). The sections were placed on copper grids and examined with TEM Jeol JEM 1010 (JEOL, Tokyo, Japan), operated at 80 kV, and coupled with a Mega View III digital camera.

To determine if the electron-dense accumulations observed inside cells were indeed CmNPs (i.e., Ag-MnO_2_NPs), the probes were examined also with a SEM Hitachi SU8230 (HITACHI, Tokyo, Japan), coupled with an EDX detector, operated at 15 kV.

### 3.8. Statistical Analyses

Each concentration was tested 12 times, and each plate contained untreated cells as positive control, negative controls (cells treated with Tween 20 at 2% concentration/well), and vehicle controls.

Data refers to mean ± S.E.M. from at least four independent experiments. Statistical analyses were performed using one-way Analysis of variance (ANOVA), Student’s *t* test, and χ^2^ test. Values of *p* ≤ 0.05 were considered significant. All calculations were performed in Origin 8 (Origin Lab Corporation, USA).

## 4. Conclusions

The plant extracts used in this study (i.e., *C. majus* and *V. minor*) facilitated the formation of uniformly distributed NPs. Three types of Ag-MnO_2_ nanoparticles with crystalline structure resulted with the use of: *C. majus* (CmNPs), *V. minor* (VmNPs), and a 1:1 mixture of the two extracts (MNPs). MNPs had an average size of 9.36 nm ± 0.19 nm, VmNPs were of 10.09 nm ± 0.14 nm, and CmNPs of 32.47 nm ± 0.73 nm. VmNPs had a balanced ratio between silver and manganese elements and negatively affected only the melanoma cells. MNPs reduced the viability of both cell lines at concentrations above 500 µg/mL, while CmNPs had a rather proliferative effect in the same experimental conditions. The characterized MNPs indicated that a 1:1 ratio of the proposed plant extracts is efficient for nanoparticle preparation, but the high content of alkaloids along with the smaller size of nanoparticles is harmful for both keratinocytes, as it is for skin melanoma cells used in this study. Thus, green synthesis provides the advantage of a good reducing agent for nanoparticle formation, with extra properties exploitable for the selective and/or targeted treatments.

The NPs prepared using only *V. minor* extract showed great potential to inhibit melanoma cells, without affecting the normal keratinocytes, MNPs negatively affected both cell lines, and CmNPs had a rather proliferative effect. The smallest and uniformly distributed NPs were MNPs, and size control is important in targeted treatment, however, further studies are required to find the optimal ratio for extract combination in order to achieve cancer cell inhibition without affecting the healthy cells. This paper showed how *V. minor* and *C. majus* used together have a great potential for NP synthesis, and in a future work the aim is to investigate the extent of damages induced in cells even when viability is not negatively affected.
